# Severe Diabetic Ketoacidosis With Extreme Acidemia and Preserved Consciousness: Intensive Care Management Guided by Pathophysiology Without Bicarbonate or Intubation

**DOI:** 10.7759/cureus.108038

**Published:** 2026-04-30

**Authors:** Orazio Stefano Giovanni Filippelli, Daniela Procopio, Anna Maria Giglio, Mario Pezzi, Stefania Faragò

**Affiliations:** 1 Anesthesia and Critical Care, Azienda Ospedaliero Universitaria ‘Renato Dulbecco’ di Catanzaro, Catanzaro, ITA

**Keywords:** conservative management, diabetic ketoacidosis, electrolyte imbalance, insulin therapy, intensive care management, kussmaul respiration, metabolic acidosis, pediatric emergency, physiology-guided management, severe acidemia

## Abstract

Diabetic ketoacidosis (DKA) is a severe and potentially life-threatening metabolic emergency, frequently observed in pediatric and adolescent patients, often representing the initial manifestation of type 1 diabetes mellitus. The presence of extreme acidemia commonly prompts aggressive interventions, including bicarbonate administration and invasive ventilatory support, although such approaches are not always supported by current evidence or the overall clinical context.

We report the case of a 16-year-old female presenting with severe DKA (pH 6.8-7.0), who was alert, oriented, and cooperative, with marked Kussmaul respiration and preserved hemodynamic stability. Lactate levels remained consistently within the normal range, effectively excluding significant systemic hypoperfusion and supporting the interpretation of a “pure” ketoacidotic state. Management included initial fluid resuscitation, followed by transfer to the intensive care unit for close monitoring and controlled metabolic correction. Continuous intravenous insulin infusion, dynamic potassium supplementation, and the timely introduction of 5% dextrose during the phase of glycemic reduction were implemented. Neither bicarbonate therapy nor endotracheal intubation was performed.

Serial arterial blood gas analyses demonstrated a progressive and coherent correction of metabolic acidosis, with closure of the anion gap within approximately 24 hours. The high-frequency monitoring strategy, including hourly glucose measurements and arterial blood gas analysis every two hours, allowed precise titration of therapy and minimized the risk of rapid osmotic shifts.

This case highlights the potential dissociation between biochemical severity and clinical presentation in DKA, emphasizing the critical role of physiological compensation. Although management was consistent with current guidelines, the combination of extreme acidemia with preserved consciousness and successful non-invasive management remains clinically uncommon and relevant. It supports a physiology-guided approach based on gradual correction and careful monitoring, rather than rapid normalization of laboratory values, thereby avoiding unnecessary and potentially harmful invasive interventions.

## Introduction

Diabetic ketoacidosis (DKA) is an acute complication of diabetes mellitus characterized by hyperglycemia, high anion gap metabolic acidosis, and accumulation of ketone bodies [1]. In pediatric and adolescent patients, it frequently represents the mode of disease onset [2].

From a pathophysiological standpoint, insulin deficiency leads to increased lipolysis and hepatic production of ketone bodies, with consequent accumulation of organic acids and development of metabolic acidosis [3]. Respiratory compensation, through hyperventilation, represents a fundamental survival mechanism, allowing a reduction in pCO₂ and a partial rebalancing of pH [3].

In addition to metabolic derangements, DKA is associated with significant fluid and electrolyte disturbances, including dehydration, sodium imbalance, and total body potassium depletion, even in the presence of normal or elevated serum potassium levels at presentation. These alterations require careful and dynamic correction, as inappropriate or overly rapid management may result in serious complications.

The main international guidelines recommend management based on gradual rehydration, continuous insulin infusion, and electrolyte correction, discouraging the routine use of bicarbonate, which is reserved for selected conditions of extreme severity [1,2]. Particular attention is given to the risk of cerebral edema, especially in pediatric patients, which has been associated with rapid osmotic shifts during treatment [4,5].

Despite these recommendations, clinical practice is often influenced by the numerical severity of acidemia, with a tendency to pursue rapid correction of pH values through aggressive interventions. However, this approach may overlook the underlying physiological compensatory mechanisms, particularly respiratory compensation, which plays a crucial role in maintaining acid-base balance.

This case underscores the importance of a pathophysiology-guided approach based on overall clinical observation, rather than the simple interpretation of numerical parameters. In particular, it highlights how extreme acidemia may coexist with preserved clinical stability when compensatory mechanisms remain effective, and how a controlled, gradual therapeutic strategy may be preferable to invasive or potentially harmful interventions. The rationale for reporting this case lies in the unusual coexistence of extreme acidemia and preserved clinical stability, allowing successful management without bicarbonate therapy or endotracheal intubation. Similar cases have been reported in the literature; however, the combination of extreme acidemia with preserved consciousness and successful non-invasive management remains relatively uncommon and clinically relevant. The case does not propose a deviation from existing guidelines, but provides a clinically relevant example of how guideline-consistent, physiology-guided management may be safely applied even in apparently extreme biochemical scenarios when compensatory mechanisms are preserved [1,2], reinforcing the importance of integrating biochemical data with clinical assessment in order to avoid unnecessary escalation of care.

## Case presentation

A 16-year-old female patient presented to the emergency department and was subsequently admitted to the Pediatric Department with a three-day history of polyuria and vomiting. She had no prior history of diabetes mellitus, and the current presentation was consistent with new-onset diabetes. At admission, she weighed 45 kg and was 155 cm tall (body mass index: 18.7 kg/m²). Laboratory evaluation documented severe DKA, with marked hyperglycemia, severe metabolic acidosis, elevated anion gap, glycosuria (>1000 mg/dL), and ketonuria (80 mg/dL). At presentation, no clear clinical focus of infection was identified. Although inflammatory markers were elevated, microbiological work-up did not document an active infection: blood cultures were negative, urine culture showed no bacterial or yeast growth, SARS-CoV-2 testing was negative, and the respiratory molecular panel did not detect bacterial or viral pathogens.

On admission, she was alert, oriented, and cooperative, with no signs of neurological impairment. Clinical assessment was dominated by marked polypnea, with a respiratory rate of approximately 30 breaths per minute, characterized by deep, regular, and labored breathing consistent with Kussmaul respiration.

Vital signs were overall preserved, with a blood pressure of 140/80 mmHg, heart rate of 130 bpm, and peripheral oxygen saturation of 99% on room air. Urine output was present and abundant. The patient reported diffuse abdominal pain, and mild abdominal tenderness was noted on physical examination; an abdominal ultrasound was performed and found to be negative, excluding a surgical cause and supporting the metabolic origin of the symptoms. Abdominal ultrasound images were not available for inclusion.

Arterial blood gas analysis revealed very severe metabolic acidosis, with pH values ranging between 6.8 and 7.0, bicarbonate levels around 1.7 mmol/L, and markedly reduced pCO₂ (approximately 7 mmHg), indicative of maximal respiratory compensation. Key laboratory findings at admission are summarized in Table 1. C-peptide levels were reduced (1.0 ng/mL), supporting endogenous insulin deficiency and consistent with new-onset type 1 diabetes mellitus.

**Table 1:** Key laboratory findings at admission The data demonstrate severe high anion gap metabolic acidosis associated with marked hyperglycemia and ketonuria, consistent with diabetic ketoacidosis. Reduced C-peptide levels support endogenous insulin deficiency, consistent with new-onset type 1 diabetes mellitus. Lactate levels within the normal range suggest the absence of significant systemic hypoperfusion.

Parameter	Result	Reference range
Blood glucose	522 mg/dL	70-100 mg/dL
pH	7	7.35-7.45
pCO₂	7 mmHg	35-45 mmHg
HCO₃⁻	1.7 mmol/L	22-26 mmol/L
Sodium (Na⁺)	125 mmol/L	135-145 mmol/L
Potassium (K⁺)	4.8 mmol/L	3.5-5.0 mmol/L
Chloride (Cl⁻)	104 mmol/L	98-107 mmol/L
Lactate	0.9 mmol/L	<2 mmol/L
Urine ketones	80 mg/dL	Negative
Creatinine	1.5 mg/dL	0.6-1.2 mg/dL
Urea	45 mg/dL	10-50 mg/dL
White blood cells	23.1 ×10³/µL	4-10 × 10³/µL
CRP	74.9 mg/L	<5 mg/L
Procalcitonin	2.26 ng/mL	<0.05 ng/mL
C-peptide	1.0 ng/mL	1.1-5.0 ng/mL
Phosphate	2.70 mg/dL	2.5-4.5 mg/dL

In the Pediatric Department, fluid therapy with normal saline was initiated at a rate of 300 mL/hour for approximately 90 minutes. Given the severity of the biochemical derangement, an intensive care consultation was requested, and the patient was transferred to the intensive care unit for continuous monitoring and controlled management of metabolic correction.

Upon admission to the intensive care unit, a urinary catheter was placed for accurate monitoring of urine output, and a radial arterial catheter was inserted for invasive blood pressure monitoring and serial arterial blood gas analysis. Following the initial phase of volume expansion, the fluid infusion rate was reduced to 170 mL/hour in accordance with a strategy of gradual rehydration.

Continuous intravenous insulin infusion was initiated within a range of 2.25 to 4.5 units/hour, corresponding to approximately 0.05-0.1 U/kg/hour, without an initial bolus. At the same time, early potassium supplementation was started and adjusted according to serial measurements, considering the total body potassium depletion typical of DKA. Serum phosphate level at admission was 2.70 mg/dL (reference range 2.5-4.5). No phosphate supplementation was required during the initial phase of treatment.

With the progressive reduction in blood glucose, 5% dextrose was introduced when values approached the range of 250-300 mg/dL, to allow continuation of insulin infusion and ongoing resolution of ketosis.

Lactate values remained consistently within normal limits (0.6-0.9 mmol/L), indicating the absence of systemic hypoperfusion and supporting the interpretation of an isolated severe DKA [3].

Despite the extreme acidemia, bicarbonate therapy was not administered, and endotracheal intubation was not performed, given the preserved neurological status and the effectiveness of respiratory compensation.

The course of serial arterial blood gas analyses documented progressive metabolic correction, with an increase in bicarbonate levels, reduction of the anion gap, and improvement in pH, leading to substantial clinical and metabolic improvement within approximately 24 hours, without complications.

The arterial blood gas and metabolic course of the patient is reported in Table 2.

**Table 2:** Serial arterial blood gas and metabolic parameters during ICU stay Time 0 corresponds to the first arterial blood gas obtained after ICU admission and arterial catheter placement. Arterial blood gases were performed every two hours, while blood glucose was monitored hourly. The reported values represent the most clinically relevant time points of the clinical course. Serial measurements show progressive correction of metabolic acidosis, with normalization of pH and reduction of the anion gap. Lactate levels remained within normal limits throughout.

Time (hour)	pH (7.35-7.45)	pCO₂ mmHg (35-45)	HCO₃⁻ mmol/L (22-26)	Na⁺ mmol/L (135-145)	K⁺ mmol/L (3.5-5.0)	Cl⁻ mmol/L (98-107)	Glucose mg/dL (70-100)	Lactate mmol/L (<2)	Anion gap mmol/L (10-20)
0 (ICU)	7	7	1.7	125	4.8	104	500	0.9	24
1.5	7.06	11	3.1	130	4.3	104	422	1	27
3	7.14	12	4.1	133	4.2	108	304	0.8	25
5	7.16	14	5	134	3.9	109	276	0.6	24
6	7.21	15	6	134	4.1	110	288	0.6	22
9	7.28	17	8	135	3.9	113	178	0.7	18
14	7.32	20	10.3	137	3.5	112	339	0.8	18
19	7.36	23	13	134	3.5	110	201	0.7	15
24	7.42	24	15.6	135	3.2	110	172	0.9	13

Arterial blood gases were obtained every two hours, while blood glucose was monitored hourly, allowing dynamic adjustment of insulin therapy and electrolyte supplementation.

Temporal analysis of the data demonstrates a progressive and coherent correction of metabolic acidosis. During the first hours, a gradual increase in bicarbonate was observed, initially from very low levels, associated with maintenance of pCO₂ values consistent with effective respiratory compensation. The reduction in the anion gap observed in subsequent phases reflects the progressive clearance of ketone bodies under the effect of insulin.

A particularly relevant element is represented by the stability of lactate levels, which remained consistently within normal limits throughout the entire course. This finding, integrated with hemodynamic stability, normal peripheral oxygen saturation, and preserved urine output, made it possible to exclude a component of systemic hypoperfusion, reinforcing the interpretation of isolated severe DKA.

The progressive reduction in blood glucose, monitored hourly, occurred in a controlled manner, allowing the timely introduction of 5% dextrose when values approached the range of 250-300 mg/dL. This allowed continuation of insulin infusion, which was essential for suppressing ketogenesis while avoiding hypoglycemia.

Overall, the temporal evolution of these parameters confirms that correction of acidosis occurred through resolution of the underlying metabolic process, without the need for exogenous buffering interventions.

## Discussion

This case highlights a marked dissociation between biochemical severity and clinical condition, emphasizing that extremely low pH values should not be interpreted in isolation as indicators of absolute clinical severity. The distinctive value of this case is not related to the use of a novel therapeutic protocol, but to the clinical context in which a guideline-consistent approach was applied [1,2]. Despite extreme acidemia, the patient remained conscious, hemodynamically stable, and able to maintain effective respiratory compensation, thereby supporting a conservative strategy without bicarbonate therapy or invasive ventilation.

In this case, symptoms suggestive of diabetes mellitus were present for only a short period (approximately three days) before the onset of DKA. Moreover, no microbiologically documented infection was identified at presentation. This combination may explain why, despite severe metabolic derangement, the patient’s general condition was initially relatively preserved.

The role of Kussmaul respiration was central in maintaining acid-base balance. Hyperventilation allows a significant reduction in pCO₂, contributing decisively to the compensation of metabolic acidosis [3]. In this context, endotracheal intubation and mechanical ventilation could have been not only unnecessary but potentially harmful, as suppression of spontaneous hyperventilation may lead to increased pCO₂ levels and worsening of both systemic and intracellular acidosis. This consideration is particularly relevant in patients who are neurologically intact and able to sustain an effective respiratory drive.

Another key element in the interpretation of this case was the persistence of lactate values within the normal range. This finding allowed exclusion of a significant component of lactic acidosis or systemic hypoperfusion, supporting the diagnosis of a “pure” ketoacidotic state. In addition to the high anion gap metabolic acidosis typical of DKA, the acid-base profile was consistent with a mixed metabolic disorder, including a component of normal anion gap metabolic acidosis. At presentation, serum chloride was within the normal range (Cl⁻ 104 mmol/L), indicating that a hyperchloremic component was not yet established. However, the presence of several days of vomiting suggests gastrointestinal bicarbonate loss as a plausible contributing mechanism. Chloride levels increased during the early phase of treatment (108 mmol/L at three hours, 113 mmol/L at nine hours) and subsequently decreased (110 mmol/L at 24 hours), consistent with the development of a transient hyperchloremic component during fluid resuscitation. The integration of this biochemical parameter with preserved hemodynamic stability and adequate urine output further reinforced the appropriateness of a conservative and physiology-guided management strategy.

The decision to withhold bicarbonate therapy is fully consistent with current guideline recommendations, which restrict its use to highly selected cases, typically characterized by extreme acidemia associated with hemodynamic instability or life-threatening hyperkalemia [1,2]. In the present case, the absence of such features, together with preserved compensatory mechanisms, supported a strategy focused on correction of the underlying metabolic disturbance rather than on rapid normalization of pH.

Management was based on gradual correction through controlled fluid resuscitation, continuous insulin infusion, and dynamic electrolyte replacement, with timely introduction of dextrose to allow continued insulin administration and effective suppression of ketogenesis. This approach reflects the core principles of DKA treatment and minimizes the risk of treatment-related complications.

Importantly, rapid osmotic shifts during DKA treatment have been associated with severe complications, including cerebral edema, particularly in pediatric populations [4,5]. The adoption of a slow and progressive therapeutic strategy, combined with high-frequency monitoring, is therefore essential to reduce this risk.

In this context, the high-frequency monitoring strategy adopted in this case (hourly blood glucose measurements and arterial blood gas analysis every two hours) allowed precise titration of insulin, fluids, and electrolytes, minimizing the risk of abrupt metabolic changes and ensuring a controlled resolution of acidosis [1,2].

The main take-home message is that extreme acidemia alone should not automatically prompt bicarbonate administration or endotracheal intubation when the patient remains neurologically intact, hemodynamically stable, and able to sustain effective respiratory compensation. In this setting, close monitoring and gradual correction of the underlying metabolic disorder may preserve physiological compensation and avoid potentially harmful interventions. Overall, this case supports a physiology-driven approach to severe DKA, demonstrating that even in the presence of extreme acidemia, invasive interventions may be safely avoided when compensatory mechanisms are preserved, and careful monitoring is ensured.

## Conclusions

Management of severe DKA should be guided primarily by pathophysiological principles rather than by the rapid normalization of laboratory values. This case illustrates that even in the presence of extreme acidemia, preserved respiratory compensation, stable hemodynamic status, and normal lactate levels may identify a subset of patients in whom a conservative and non-invasive approach is both safe and effective. This is supported by the progressive normalization of arterial blood gas parameters and anion gap observed during the clinical course. The findings emphasize the central role of compensatory mechanisms, particularly Kussmaul respiration, which should be recognized and preserved rather than suppressed. In this context, premature or unnecessary invasive interventions, such as bicarbonate administration or endotracheal intubation, may disrupt physiological balance and potentially worsen the metabolic condition. A gradual therapeutic strategy based on controlled fluid resuscitation, continuous insulin infusion, and careful, dynamic electrolyte management allows resolution of the underlying metabolic disturbance while minimizing the risk of treatment-related complications. High-frequency monitoring further supports safe titration of therapy and helps prevent rapid osmotic shifts. Overall, this case reinforces the importance of a physiology-driven approach to severe DKA, highlighting that careful observation and controlled correction may be preferable to aggressive interventions, even in apparently extreme biochemical scenarios. In selected patients with preserved consciousness and effective respiratory compensation, extreme acidemia alone should not be considered an absolute indication for endotracheal intubation or bicarbonate therapy.
